# What he knows about her and how it affects her? Husband’s knowledge of pregnancy complications and maternal health care utilization among tribal population in Maharashtra, India

**DOI:** 10.1186/s12884-019-2214-x

**Published:** 2019-02-13

**Authors:** Suresh Jungari, Balram Paswan

**Affiliations:** 1 Interdisciplinary School of Health Sciences, Saritribai Phule Pune University, Pune, Maharashtra 411007 India; 2 Department of Population Policies and Programmes, International Institute for Population Sciences, Mumbai, India

**Keywords:** Husbands’ awareness, Maternal health care utilization, Tribal women, Complication knowledge

## Abstract

**Background:**

*Husbands’* knowledge and awareness of pregnancy complications have a positive impact on their wives’ utilization of maternal health care services. In this study, we examined whether husbands’ knowledge and awareness of pregnancy complications can serve as determinants of maternal health service utilization among wives from the tribal population.

**Methods:**

This cross-sectional study was conducted in the rural Gadchrioli district of Maharashtra, India, during November 2014–March 2015. This study included a representative population-based sample of 385 men whose wives had given birth in last 2 years at the age of 15–49 years. A multistage sampling strategy was adopted to select the respondents. Univariate, bivariate, and binary logistic regression analyses were applied to examine the association between men’s knowledge and maternal health service utilization.

**Results:**

The result revealed that an increase in husbands’ education level increased the wives’ utilization of antenatal (ANC) care services. The type of tribe also contributed to significant differences in ANC utilization (OR: 2.64; 95% CI: 0.847–8.24). Regarding standard of living, husbands who were poor were 22% less likely than husbands in the rich category to report the utilization of ANC by their wives. Men with partial or complete knowledge of pregnancy, childbirth, and postpartum complications were more likely to utilize all maternal health services by their wives.

**Conclusions:**

The wives are of men who aware of complications during pregnancy and childbirth are more likely to use maternal health services. Therefore, educating and empowering men about pregnancy complications will contribute to the reduction in maternal and neonatal deaths.

**Electronic supplementary material:**

The online version of this article (10.1186/s12884-019-2214-x) contains supplementary material, which is available to authorized users.

## Background

Every day in 2015, approximately 830 women died due to complications during pregnancy and childbirth. Almost all of these deaths occurred in low-resource settings, and most could have been prevented [1]. The risk of a woman in a developing country dying from maternal-related causes during her lifetime is approximately 33 times higher than a woman in a developed country. Many determinants account for safe motherhood. The utilization of maternal health services such as complete antenatal care, institutional delivery services, and postnatal care services is associated with multiple factors that influence, directly or indirectly, the health of the mother and infant. In developing countries, maternal health service utilization is poor compared with that in developed countries. In India, maternal health service utilization is lower among tribal communities than among other social groups [2–5]. The reasons for the underutilization of maternal health services differ from place to place in the country. The major reasons are the distance from a health facility, unavailability, cultural barriers, educational level of women, economic inequalities, household status, and other demographic factors [6, 7]. The reproductive and child health programs of India exclusively focus on women to increase maternal health service utilization.

To improve maternal health, reproductive and child health programs began to focus on the male partner in 1990, and reproductive health concerns have gained continual importance. Many studies have reported the positive benefits of male knowledge of pregnancy complications for women health in developed and developing countries; these benefits include the following: the increased use of antenatal services, institutional delivery services, and postnatal care [8–10]. Male participation and support for women during pregnancy also discourage unhealthy practices such as smoking and tobacco use among women [11, 12].

In India, maternal mortality declined from 240 maternal deaths for 100,000 live births in 2004 to 176 maternal deaths for 100,000 live births in 2016. However, the desired level of decline has not been achieved, and the decline is not uniform across states and social groups. Scheduled caste (SC) and scheduled tribal (ST) communities have higher maternal mortality and morbidity than other social groups in India [13, 14]. The tribal population has far worst health indicators due to the underdevelopment, remote living conditions, lack of availability and accessibility maternal health services.

Husbands with knowledge of complications during pregnancy and delivery are more likely to promote wives for seeking antenatal care services, institutional deliveries, and postnatal services. Further, knowledgeable husbands will contribute to the birth preparedness [15–17]. In countries such as India, the majority of decisions related to pregnancy and childbirth, including the place of delivery, are taken by a male member of the household or the husband; therefore, well-informed men will make positive decisions for women [18, 19]. Several studies have provided sufficient evidence of positive childbirth outcomes for male involvement in maternal health. However, husbands’ knowledge of pregnancy complications and its association with maternal health service utilization have not been specifically documented in the Indian literature on maternal health. Furthermore, tribal men’s level of knowledge of pregnancy complications has been rarely studied. Therefore, in this study, we examined the men’s knowledge and awareness of pregnancy complications and maternal health service utilization among the Gond and Madiya tribal communities in Maharashtra, India.

## Methods

### Data source

This study was conducted in the Gadchiroli district of Maharashtra, India, from November 2014 to March 2015. The estimated population of Gadchiroli district is 1,072,942, with the male population being 541,328 and the female population being 531,614 as per the 2011national census. In 2011, the literacy rate among men was 82.31%. Paddy is the main agricultural produce in the district. The other agricultural produces in the district are jwar, linseed, tur, and wheat. The district has comprises 12 administrative blocks. The district as 1 civil hospital, 13 rural hospitals, 45 primary health centers, and 36 primary health units.

### Study design and sampling

This study was designed as a cross-sectional descriptive community survey. A multistage sampling procedure was employed to select the participants of the study. In the first stage, the district was selected purposively. In the second stage, the block was selected on the basis of the highest tribal population. In the third stage, 19 villages were selected using the probability proportional to size sampling method. From these villages, a list of women who delivered in the last 2 years was collected from local health care providers. In total, 385 men were interviewed those wives have given birth in last 2 years. The study excluded the men those wives died during childbirth or postpartum period. The response rate for this study was 95%.

### Variable description

Face to face interviews were conducted to collect the data. Before data collection pretest was conducted on 10% sample of study in the same areas were study carried out. The questionnaire included questions related to socio-demographic characteristics such as age, education level, standard of living of household, and sanitation facilities. To capture the men’s awareness and knowledge about pregnancy complication we have adopted the safe motherhood questionnaire developed by the Maternal and Neonatal Program of JHPIEGO, an affiliate of John Hopkins University was used with some modifications with local context [20]. (See Additional file 1) This tool is widely used in the low resource setting to assess the pregnancy complications knowledge among both men and women [21, 22]. The participants’ awareness and knowledge of complications during pregnancy, during delivery, and after delivery were determined. Possible complications during pregnancy included vaginal bleeding, convulsions, prolonged labor, fever for 3 days, hypertension, no movement of the fetus, and urinary problems. Possible complications during delivery included excessive vaginal bleeding, ruptured uterus, premature labor, prolonged labor, breech presentation, and weakness. Possible complications in the postpartum period were excessive vaginal bleeding, convulsions, high fever, lower abdominal pain, vaginal discharge, painful urination, depression, severe headache, and weakness.

### Outcome variables

Antenatal Care: Any antenatal care taken by women during pregnancy.

Place of delivery: The study reported institutional delivery or home delivery.

Post Natal Care: Any postnatal care taken by women after delivery.

All three variables were used as outcome variables, which are presented as dichotomous variables.

### Independent variables

Men’s knowledge and awareness were assessed in three areas: complications during pregnancy, during childbirth, and in the postnatal period. The questionnaire contained multiple choice questions for each area. Each answer was coded as yes or no. Using these multiple choice questions, we created an index of three categories: no knowledge, partial knowledge, and complete knowledge of complications during pregnancy, during delivery, and after delivery. Those who reported that they did not know of any complication were coded as no knowledge, those who reported that they know of two complications were coded as partial knowledge, and those who reported that they know of more three complications and more were coded as complete knowledge. This categorization was made based on previous studies [23–25]. The psychometric properties for the above questions calculated based on Cronbach’s alpha value.

### Other variables

In this study, the other key variables were the respondents’ age, current occupation, type of marriage (arranged or love), type of tribe (Gond or Madiya), education level (illiterate, primary, secondary, or graduation and above), type of family (joint or family), media exposure (high, moderate, or low), wealth index (low, medium, or high).

### Statistical analysis

Data collected were coded and entered into CSPro software and were exported to SPSS, version 22, for analysis. Frequency, percentage, and descriptive summaries were used to describe the study variable by using univariate analysis. Logistic regression was performed to identify the association of knowledge of pregnancy complications with maternal health service utilization. These results are expressed as odds ratio (OR) and 95% confidence interval (CI). *P* value 0.05 denoted a significant association between outcome and explanatory variables.

### Ethical concerns

The Institutional Review Board of the International Institute of Population Sciences, Mumbai, India, reviewed the study and granted ethical approval. Permission to conduct this study was granted by the local leaders of Gond and Madiya tribes and village heads. Respondents were provided an explanation of the study objectives and rationale; they were informed of their right to stop the interview at any time if they wished, without giving any reason. They were also told that their names would not be revealed anywhere. Information collected from individuals and the community was used for only academic research purposes.

## Results

### Socio-demographic characteristics of respondents

Table 1 presents the socio-demographic profile of the respondents. Nearly 41% of respondents belonged to the age group of 18–25 years, 43% to the 26–30 years age groups, 12% were aged 31–35 years. Around 28 of the respondents were illiterate. About 18%had completed primary schooling, 38% had studied up to secondary level education.

**Table 1:** Socio-demographic characteristics of the respondents

Background characteristics	Cases (N)	Percent
Current age		
19–24	95	24.7
25–29	169	43.9
> 29	121	31.4
Education		
Illiterate	108	28.1
Primary	70	18.2
Secondary	147	38.2
Higher secondary	60	15.6
Type of family		
Nuclear family	234	60.8
Joint family	151	39.2
Type of marriage		
Arrange marriage	295	76.6
Love marriage	90	23.4
Type of tribes		
Gond	142	36.9
Madiya	243	62.9
Current Occupation		
Agriculture	213	57.0
Agricultural labour	91	24.3
Government/Private employees	55	14.7
Others	15	4.0
Media exposure		
No exposure	152	65.5
Any exposure	133	34.5
Total	385	100

Agriculture is the dominant occupation with 57% of the respondents being engaged in it followed by agriculture labourer (24%) and employment in the government and private sector (14% of the sample). Around 60% of the families were nuclear with the remaining being joint. There were only two tribal groups living in the study area, the Gonds and Madiyas who comprised of 36 and 67%. Nearly 23.4% of the men had opted for love marriages. More than 75% respondents had an arranged marriage.

### Men’s knowledge pregnancy complications

Table 2 shows knowledge about complications during pregnancy, delivery and after childbirth. Nearly 40% of the respondents reported that they did not know of any complications during pregnancy, childbirth and postpartum. Most of the men (58%) reported that they were aware of convulsions during pregnancy, prolonged labour (42%) fever (31%) and vaginal bleeding (24%). Regarding complications during pregnancy, less than one third of the respondents reported awareness of excessive bleeding, premature labour and prolonged labour. About 27% men reported that they knew about ruptured uterus and weakness. Only half of the respondents said that they had knowledge about complications during delivery. From the Table 2, it is seen that 47.8% of the men had no knowledge about the possible complications after delivery. Most of them (51%) said that women experience weakness after childbirth, and 42% said that the women have lower abdominal pain. About 28% of the men also said that women have high fever and 15% said that they were aware of depression.

**Table 2:** Percentage of men’s knowledge of complications during pregnancy, delivery and postnatal period

Complications	Percent	Cases (N)
Vaginal bleeding	24.7	95
Convulsions	58.7	226
Prolonged labour	42.6	164
Fever for three days	31.7	122
Hypertension	21.6	83
No movements of fetus	17.4	67
Urinary problems	15.6	60
Don’t know	39.5	152
Complications during delivery		
Excessive bleeding	31.4	121
Ruptured uterus	26.8	103
Premature labour	30.9	119
Prolonged labour	31.4	121
Breech presentation	23.4	90
Weakness	27.9	107
Don’t know	49.9	165
Complications after delivery		
Excessive bleeding	16.4	63
Convulsions	17.9	69
High fever	28.3	109
Lower abdominal pain	42.3	163
Vaginal discharge	18.7	72
Painful urination	15.6	60
Depression	16.1	62
Severe headache	23.4	90
Weakness	51.2	197
Don’t know	47.8	184

Multiple answers

### Respondents’ socio-demographic characteristics and maternal health care service utilization

Socio-demographic characteristics and maternal health service utilization are presented in Table 3.Men aged 18–25 years reported higher utilization of ANC, institutional delivery, and PNC services. With the increasing age of respondents, the use of all three-maternal health services decreased. From the table, it is evident that the increasing education level of men contributed to the increased use of all three maternal health care services, and their education level was positively associated with health care service utilization. Regarding the occupation of men, men employed in salaried jobs reported higher utilization of maternal health services for their wives. Husbands’ type of job was statistically significantly associated with the utilization of all maternal health services. However, the type of family was not significantly associated with maternal health service utilization. Regarding the type of marriage, men who had a love marriage to their wives showed higher utilization of all three domains of maternal health services. Men in the rich category of the wealth index showed high utilization of all three domains of maternal health services for their wives.

**Table 3:** Percentage utilization maternal health care services by background characteristics of husbands

Background characteristics	Percent of Utilization of ANC (n)	Percent of Institutional Delivery(n)	Percent of Utilization of PNC (n)
Current age			
19–24	97.9(93)	48.4(46)	55.8(53)
25–29	95.9(162)	51.5(87)	63.9(108)
> 29	94.2(114)	44.6(54)	47.9(58)
Education			
Illiterate	88.0(95)	26.9(29)	34.3(37)
Primary	97.1(68)	45.7(32)	44.3(31)
Secondary	99.3(146)	57.1(84)	66.7(98)
Graduation and above	100(60)	70.0(42)	88.3(53)
Current Occupation			
Agriculture	96.2(205)	43.7(93)	55.9(119)
Agriculture labours	93.4(85)	44.0(40)	45.1(41)
Govt./Private employees	98.2(54)	65.5(36)	76.4(42)
Others	96.2(25)	69.2(18)	65.4(17)
Type of family			
Nuclear family	96.2(225)	51.3(120)	60.7(142)
Joint family	95.4(144)	44.4(67)	51.0(77)
Type of tribes			
Gond	94.4(134)	54.2(77)	62.0(88)
Madiya	96.7(235)	45.3(110)	53.9(131)
Type of marriage			
Arrange marriage	95.3(281)	45.8(135)	50.8(150)
Love marriage	97.8(88)	57.8(52)	76.7(69)
Wealth Index			
Rich	98.4(126)	66.4(85)	70.3(90)
Middle	96.9(125)	35.7(46)	51.2(66)
Poor	92.2(118)	43.8(56)	49.2(63)
knowledge of complications during pregnancy			
No knowledge	93.4(142)	35.5(54)	36.2(55)
Partial knowledge	95.9(139)	49.7(72)	63.4(92)
Full knowledge	100.0(88)	69.3(61)	81.8(72)
knowledge of Complications during delivery			
No knowledge	94.5(156)	36.4(60)	35.8(59)
Partial knowledge	97.6(123)	46.8(59)	69.0(87)
Full knowledge	95.7(90)	72.3(68)	77.7(73)
knowledge of Complications after delivery			
No knowledge	93.5(172)	35.3(65)	31.5(58)
Partial knowledge	97.6(120)	52.0(64)	74.8(92)
Full knowledge	98.7(77)	74.4(58)	88.5(69)
Total	95.8(369)	48.6(187)	56.9(219)

### Regression results of men’s knowledge of complications during pregnancy and maternal health service utilization

Table 4 shows logistic regression models that were used to examine the association between men’s knowledge and maternal health service utilization. Model 1 showed the association between socio-demographic variables and ANC utilization, model 2 examined the association between husbands’ levels of knowledge of pregnancy complications and maternal ANC service utilization. Model 3 showed the association between both socio demographic variables and pregnancy complication knowledge of husbands and utilization of ANC services for their wives.

**Table 4:** Result of logistic regression showing antenatal care utilization by selected background characteristics and husbands knowledge of pregnancy complications

Background characteristics	Model 1			Model 2			Model 3		
	AOR	95 %CI		AOR	95 %CI		AOR	95 %CI	
Current age									
19–24®									
25–29	0.36	0.060	2.187				0.466	0.071	3.054
> 29	0.53	0.091	3.148				0.434	0.069	2.736
Education									
illiterate®									
Primary	5.62**	1.082	29.169				8.757**	1.286	59.613
Secondary	5.758***	2.590	12.800				5.758***	2.590	215.439
Graduation and above	9.547***	2.980	33.689				9.473***	2.746	32.042
Current Occupation									
Agriculture®									
Agriculture labours	1.18	0.320	4.379				0.868	0.205	3.676
Govt./Private employees	0.730	0.073	7.264				0.558	0.047	6.625
Others	0.549	0.055	5.438				0.392	0.031	4.904
Type of family									
Nuclear family®									
Joint family	0.754	0.242	2.347				0.585	0.161	2.122
Type of tribes									
Gond®									
Madiya	2.64*	0.847	8.249				3.687**	0.989	13.751
Type of marriage									
Arrange marriage®									
Love marriage	1.12	0.220	5.762				0.579	0.093	3.615
Wealth Index									
Rich®									
Middle	0.720	0.122	4.263				0.403	0.042	3.913
Poor	0.22*	0.040	1.203				0.137*	0.014	1.304
knowledge about complications during pregnancy									
No knowledge®									
Partial knowledge				7.09*	0.765	65.656	10.324	0.440	242.140
Complete knowledge				9.12	1.946	72.392	10.786	0.56	78.50
knowledge about complications during delivery									
No knowledge®									
Partial knowledge				0.215	0.018	2.626	0.046	0.001	1.658
Complete knowledge				0.031**	0.002	0.411	0.006**	0.000	0.261
knowledge about complications after delivery									
No knowledge®									
Partial knowledge				3.88*	0.812	18.503	5.028	0.704	35.935
Complete knowledge				5.16	0.465	57.233	3.507	0.196	62.870

Note: ® is the reference category, level of significance * *p*-value of < 0.05, **p-value of < 0.01, ***p-value of < 0.001

In Model 1, husband-related characteristics were considered; in Model 2, husband’s knowledge of pregnancy complications was added; in Model 3, two sets of variables were considered. The results indicated a statistically significant association between husbands’ socio-demographic characteristics and wives’ utilization of ANC services. The result revealed that an increase in husbands’ education level increased the wives ‘utilization of ANC care services (OR: 5.75; 95% CI: 2.590–12.800). The type of tribe also contributed to significant differences in ANC utilization (OR: 2.64; 95% CI: 0.847–8.24). Regarding standard of living, husbands who were poor were 0.22 times less likely than husbands in the rich category to report the utilization of ANC by their wives.

In Model 2, husbands ‘knowledge of pregnancy was included. In this model, it was found that husbands with partial knowledge of pregnancy complications reported higher utilization of ANC by their wives than husbands having no knowledge (OR: 7.09; 95% CI: 0.76–65.65). Thus, husbands’ partial knowledge of complications during delivery was associated with their wives utilization of ANC services. By contrast, husbands with partial knowledge of complications after delivery were 3 times more likely to report the utilization of ANC by their wives than husbands having no knowledge of complications after delivery (OR: 3.88; 95% CI: 0.812–18.503). In Model 3, husbands’ characteristics and their knowledge of pregnancy were considered. It was found that husbands’ education level, Madiya type of tribe, husbands in the poor category, and complete knowledge of complications during delivery in Model 3 had same significance as those in Model 1 and Model 2.

Regression analysis was applied to understand the adjusted effect of all selected variables on the place of delivery. In Table 5, model 1 was adjusted for the socio-demographic characteristics of husbands. It was found that the education level, occupation, and wealth index were statistically significant determinants of the place of delivery for their wives. Moreover, husbands with primary education, secondary education, and high school education and more were 2.29, 3.86, and 5.75 times, respectively, more likely to report wives’ institutional delivery than husbands who were illiterate. Husbands engaging in other occupations were more likely to report their wives’ institutional delivery (OR: 2.38; 95% CI: 0.914–6.226) than their counterpart. According to the wealth index, husbands in the middle and poor categories were 0.32 and 0.52 times, respectively, less likely to report their wives’ institutional delivery. In Model 2, all background variables were excluded, and only husbands’ knowledge of pregnancy was included. After adjusting for husbands’ knowledge of pregnancy, their knowledge of complications after delivery was statistically significantly associated with the place of delivery. Husbands ‘with partial knowledge and those with complete knowledge were 1.65 (95% CI: 0.962–2.85) and 3.66 (95% CI: 1.83–7.32) times, respectively, more likely to report wives’ institutional delivery than those having no knowledge. Furthermore, the results for Model 3 were similar to this for Model 1 and Model 2; specifically, husbands’ education level, occupation, wealth index, and knowledge of complications after delivery significantly determined their wives’ institutional delivery.

**Table 5:** Result of logistic regression showing utilization of institutional delivery by selected background characteristics and husbands knowledge of pregnancy complication

Background characteristics	Model 1			Model 2			Model 3		
	AOR	95 %CI		AOR	95 %CI		AOR	95 %CI	
Current age									
19–24®									
25–29	1.340	0.765	2.348				0.713	0.375	1.356
> 29	1.448	0.776	2.699				0.944	0.543	1.643
Education									
Illiterate®									
Primary	2.296**	1.149	4.589				0.330	0.134	0.809
Secondary	3.865***	2.129	7.015				0.766	0.304	1.932
Graduation and above	5.758***	2.590	12.800				1.085	0.496	2.373
Current Occupation									
Agriculture®									
Agriculture labours	1.418	0.807	2.492				0.395*	0.144	1.079
Govt./Private employees	1.382	0.665	2.875				0.794	0.270	2.331
Others	2.386*	0.914	6.226				0.601	0.189	1.909
Type of family									
Nuclear family®									
Joint family	0.781	0.494	1.235				1.152	0.717	1.853
Type of tribes									
Gond®									
Madiya	0.745	0.462	1.202				1.178	0.717	1.937
Type of marriage									
Arrange marriage®									
Love marriage	0.986	0.566	1.716				1.113	0.624	1.983
Wealth Index									
Rich®									
Middle	0.323***	0.186	0.562				1.787**	0.993	3.216
Poor	0.522**	0.298	0.913				0.614*	0.348	1.083
knowledge of complications during pregnancy									
No knowledge®									
Partial knowledge				1.486	0.631	3.504	1.353	.518	3.533
Complete knowledge				1.519	0.573	4.028	1.290	.436	3.821
knowledge of complications during delivery									
No knowledge®									
Partial knowledge				0.736	0.295	1.836	0.566	0.204	1.567
Complete knowledge				1.846	0.686	4.966	1.707	0.576	5.060
knowledge of complications after delivery									
No knowledge®									
Partial knowledge				1.658*	0.962	2.858	1.672***	.913	3.064
Complete knowledge				3.665***	1.835	7.321	2.639	1.194	5.830

Note: ® is the reference category, level of significance * *p*-value of < 0.05, **p-value of < 0.01, ***p-value of < 0.001

The results of regressions analysis of PNC service utilization are presented in Table 6. Model 1 was adjusted for husbands’ background characteristics such as age, education level, occupation, type of family, type of tribe, and wealth index. In this model, four factors statistically significantly determined the utilization of postnatal care by respondents’ wives. For example, husbands with secondary education and high school education and more were 3 and 10 times, respectively, more likely to report wives’ postnatal utilization than those in the reference category. According to the type of family, husbands living in a joint family were less likely to utilize postnatal care (OR: .63; 95% CI: 0.395–1.013) than those living in a nuclear family. Husbands with a love marriage were 1.8 times more likely to report wives’ postnatal utilization than those with an arranged marriage. According to the wealth index, husbands in the middle and poor categories (OR: .580; 95% CI: 0.328–1.025 and OR: 0.608; 95% CI: 0.338–1.095, respectively) were less likely to report their wives’ postnatal utilization than those in the rich category.

**Table 6:** Result of logistic regression showing postnatal care utilization by selected background characteristics and husbands knowledge of pregnancy complications

Background characteristics	Model 1			Model 2			Model 3		
	AOR	95 %CI		AOR	95 %CI		AOR	95 %CI	
Current age									
19–24®									
25–29	1.618	0.898	2.915				1.813*	0.946	3.477
> 29	1.038	0.549	1.963				1.012	0.506	2.021
Education									
Illiterate®									
Primary	1.364	0.699	2.661				1.112	0.535	2.312
Secondary	3.245***	1.830	5.754				2.026**	1.073	3.823
Graduation and above	10.315***	4.003	26.578				4.765**	1.630	13.930
Current occupation									
Agriculture®									
Agriculture labours	0.810	0.461	1.423				1.481	0.792	2.770
Govt./Private employees	1.086	0.484	2.437				1.048	0.423	2.599
Others	0.971	0.370	2.544				0.921	0.312	2.713
Type of family									
Nuclear family®									
Joint family	0.633*	0.395	1.013				0.745	0.446	1.245
Type of tribes									
Gond®									
Madiya	0.666	0.407	1.089				0.883	0.515	1.515
Type of marriage									
Arrange marriage®									
Love marriage	1.824*	0.995	3.347				1.739	0.895	3.379
Wealth index									
Rich®									
Middle	0.580*	0.328	1.025				0.695	0.373	1.295
Poor	0.608*	0.338	1.095				0.845	0.443	1.612
knowledge of complications during pregnancy									
No knowledge®									
Partial knowledge				1.442	0.581	3.583	1.624	0.630	4.186
Complete knowledge				2.424	0.826	7.112	2.148	0.684	6.741
knowledge of complications during delivery									
No knowledge®									
Partial knowledge				1.290	0.490	3.395	1.049	0.383	2.877
Complete knowledge				1.126	0.387	3.272	0.905	0.294	2.787
knowledge of complications after delivery									
No knowledge®									
Partial knowledge				4.843***	2.731	8.587	4.837***	2.571	9.101
Complete knowledge				10.172***	4.403	23.500	7.161***	2.854	17.968

Note: ® is the reference category, level of significance * *p*-value of < 0.05, **p-value of < 0.01, ***p-value of < 0.001

The results indicated a statistically significant association between wives’ PNC utilization and husbands’ knowledge of pregnancy complications in Model 2. Husbands with partial knowledge and complete knowledge of complications after delivery (OR: 4.843; 95% CI: 2.731–8.587 and OR: 10.17; 95% CI: 4.403–23.50, respectively) were more likely to report wives’ PNC utilization than their counterpart. The results for Model 3, which included both husbands ‘characteristics and knowledge of pregnancy, husbands’ education level and knowledge of complications after delivery were similar to Model 1 and Model 2. In addition, husbands aged 25–29 years were 1.81 times more likely to report their wives’ postnatal check than in those in reference category.

## Discussion

In this study, Gond and Madiya tribal men’s level of knowledge and awareness of complications during pregnancy, during childbirth, and in the postnatal period were found to be poor. A large proportion of men were not aware of at least one pregnancy complication. Furthermore, illiterate men were less likely to possess knowledge of complications during pregnancy, during childbirth, and in the postpartum period. Awareness of the complications of vomiting, abdominal pain, and convulsions was high among the study participants. Nearly half of the tribal men were unaware of any pregnancy complication during pregnancy. This finding is consistent with the findings of previous studies conducted in South Asia and India [26]. Tribal people in India live in remote areas. They are not exposed to development; thus, overall development is extremely poor.

The major results of this study are the association found between husbands’ knowledge of pregnancy complications and maternal health care service utilization. Husbands with complete knowledge of pregnancy complications were more likely to report antenatal care, delivery, and postnatal care utilization by their wives than husbands having no knowledge. To the best of our knowledge, this is the first study to examine the relationship between men’s knowledge of pregnancy complications and maternal health care utilization among tribal communities in India. Studies have identified a wide range of factors responsible for maternal health service utilization in India. These factors include the limited availability of health care within an area, lack of health care human resources, poor road connectivity, poor level of awareness of institutional delivery services, and lack of transportation [27–30]. National and sub national studies have reported that the knowledge of pregnancy complications among men or husbands have positive health outcomes for women in the general population. Male support and involvement in the matters of reproductive health of women also have positive outcomes for women health, specifically for maternal health care service utilization [19, 31–34]. Moreover, men with higher levels of awareness related to pregnancy complications are more likely to participate in maternal health.

Most studies have been conducted in women to evaluate the knowledge of pregnancy complications; however, to the best of our knowledge, this study is the first to examine husbands’ knowledge of pregnancy complications and its association with maternal health care service utilization by wives. The main study rationale was that men are the primary decision makers regarding health and health care in the family and also regarding reproductive health concerns. Therefore, educating the male population about concerns and problems of maternal health in general and pregnancy complications in particular is an important strategy to achieve the higher utilization of maternal health services.

The study results are significantly useful for policy makers, program managers, and health care workers in addressing maternal health concerns, specifically those in underserved populations such as tribal communities in India. Increasing the education levels of the tribal population is an urgent requirement. Utilizing information, education and communication (IEC) material to demonstrate the causes and symptoms of pregnancy complications to communities may be an effective strategy for increasing the education levels. Further, educating men about pregnancy complications could be important component of any intervention to address the maternal health.

### Study limitations

This study has several limitations. First, the study sample is smaller than that of other national studies. This study was conducted in two specific tribal groups, namely Gond and Madiya, in the tribal-dominated district of Gadchiroli, Maharashtra, India. Therefore, the results of this study may not by generalizable to the general population or other tribal groups. This study examined men’s knowledge of complications during pregnancy, during childbirth, and in the postpartum period and its association with maternal health service utilization; however, other factors may play an important role in maternal health service utilization; this is a major limitation of this study. This study was conducted in men whose wives had given birth in last 2 years; occasionally, another person in the family may be aware of pregnancy complications, eventually leading to the use of maternal health services. While conducting interviews with men, researchers provided the possible answers in options so that they could immediately match their awareness to particular options; therefore, it is possible that respondents provided socially acceptable answers.

Since the use of maternal services occurs before the survey of men, that use may influence the men’s knowledge of complications, especially if the wives of had any complications. The does not received any specific education from any health care workers. The men’s knowledge of complications entirely dependent on the men’s interest to learn from local health workers such as ASHAs and Multipurpose workers.

## Conclusion

Despite these limitations, the findings of the study are useful for program managers and health care providers. Engaging and informing husbands about pregnancy and pregnancy complications and the importance of maternal health service use comprise a useful strategy to reduce maternal mortality and morbidity in the tribal population. Community health care workers play an important role in informing husbands about pregnancy-related matters; this represents an important strategy that can lead to safer deliveries.

## Additional file

Additional file 1: The questionnaire used for this study to assess the men’s knowledge of pregnancy complications and utilization of maternal health care services by their wives. Brief description of the data: Questionnaire examining the men’s knowledge of pregnancy complications and utilization of maternal health care services by their wives. (DOCX 25 kb)

## Abbreviations

ANC: Antenatal care
ASHA: Accredited Social Health Activist
PNC: Postnatal care
