# Supplementary material for: What he knows about her and how it affects her? Husband’s knowledge of pregnancy complications and maternal health care utilization among tribal population in Maharashtra, India
Source: BMC Pregnancy Childbirth. 2019 Feb 13;19:70. doi: 10.1186/s12884-019-2214-x (PMC6373054; doi:10.1186/s12884-019-2214-x)
Supplement: Supplementary file 1 — The questionnaire used for this study to assess the men’s knowledge of pregnancy complications and utilization of maternal health care services by their wives. Brief description of the data: Questionnaire examining the men’s knowledge of pregnancy complications and utilization of maternal health care services by their wives. (DOCX 25 kb) [file 12884_2019_2214_MOESM1_ESM.docx]

**Male Questionnaire**

| **(A)Demographic and other particulars of household members (Household roster)** | | | | | | | | |
| --- | --- | --- | --- | --- | --- | --- | --- | --- |
| Line No | Name of the member  start with the head of HH | Relation to head | Sex | Age | Marital status | Age at marriage | Education  statue | Occupation |
|  | Q.1 | Q.2 | Q.3 | Q.4 | Q.5 | Q.6 | Q.7 | Q.8 |
|  |  |  |  |  |  |  |  |  |
|  |  |  |  |  |  |  |  |  |
|  |  |  |  |  |  |  |  |  |
|  |  |  |  |  |  |  |  |  |
|  |  |  |  |  |  |  |  |  |
|  |  |  |  |  |  |  |  |  |

| **Code for block B** | | |
| --- | --- | --- |
| **Code for Q 2**  Relationship to HH  1= Household head/self  2= Husband/ Wife  3=Son/ daughter  4= Daughter in –law  5= Grandson /daughter  6= Father/ mother  7= Father in-law/ Mother in-law  8= Sister/ brother | **Code for Q 5**  1=Married  2= Widows  3= Separate/ Divorced  4= Unmarried | **Code for Q 7**  1= Illiterate  2= Primary  3=Secondary  4= High school  5= Graduate and above |
|  | **Code for Q.8**  1= Agriculture  2= Employer  3= Casual labour  4= Own business  5= Others |  |

| **Household Questionnaire** | | | | |
| --- | --- | --- | --- | --- |
| **(B) Househlold Characteristics** | | | | |
| **Sr. No.** | **Question and filter** | **Coding Category** |  | **Skip** |
| B.1 | Type of family | Nuclear family  Joint family | 1  2 |  |
| B.2 | From which tribal community you belongs? | Gond  Madiya  Rajgond  Others | 1  2  3  4 |  |
| B.3 | Does your household own any of the following?  (**Multiple responses**) | Cot  Presser cooker  Electric fan  Radio or transistor  Television  Sewing machine  Land-line/mobile telephone  Computer/Laptop  Refrigerator  Watch/Clock  Bicycle  Motor cycle  Bullocks  Other specify ( ) | Y  1  1  1  1  1  1  1  1  1  1  1  1  1 | No  2  2  2  2  2  2  2  2  2  2  2  2  2 |
| B.4 | Does your household own any of the following? | Cows/Bulls/Buffaloes  Horses/Donkeys/Mule  Goats  Sheep  Chickens/Ducks  Piggery  Others | Yes  1  1  1  1  1  1 | No  2  2  2  2  2  2 |
| **Male questionnaire**  **Background Characteristics**   \| C.1 \| How old you at your last birthday? \| \|  \|  \| \| --- \| --- \|   Age in completed years. \|  \| Skip \| \| --- \| --- \| --- \| --- \| --- \| --- \| --- \| \| C.2 \| Year of schooling you have completed? \| \|  \|  \| \| --- \| --- \|   Completed years \|  \|  \| \| C.3 \| Are your engaged in work? \| Yes  No \|  \|  \| \| C.4 \| What type of work you are doing now? \| Do not work  Agriculture  Agriculture labor  Govt. service  Private job  Manual worker  Own business  Household and domestic  Other (specify \| 1  2  3  4  5  6  7  8  9 \|  \| \| C.5 \| What was your age at the time of your marriage? \| In completed year \|  \|  \| | | | | |
| \| **(D) Information on knowledge of complications during pregnancy, delivery and postpartum** \| \| \| \| \| \| --- \| --- \| --- \| --- \| --- \| \| Sr. No. \| **Question and filter** \| **Coding Category** \|  \| **Skip** \| \| D.1 \| Do you know about pregnancy complications? \| Yes  No \| 1  2 \|  \| \| D.2 \| If you know pregnancy complication, what are the complications which can occur during pregnancy  (**Multiple responses**) \| Vaginal bleeding?  Convulsions?  Prolonged labour  Fever for 3 days  Hypertension  No movement of fetus  Urinary problems  Others \| A  B  C  D  E  F  G  H \|  \| \| D.3 \| Do you aware about the compilations during delivery \| Yes  No \| 1  2 \|  \| \| D.4 \| What are the possible problems women may face during delivery?  (**Multiple responses**) \| Excessive bleeding  Ruptured uterus  Premature labour  Prolonged labour  Breech presentation  Weakness  Any other ( ) \| A  B  C  D  E  F  G \|  \| \| D.5 \| Do you aware about postnatal care? \| Yes  No \| 1  2 \|  \| \| D.6 \| According to you what are the problem women face after delivery?  (**Multiple responses**) \| Excessive bleeding  Convulsions  High fever  Lower abdominal pain  Vaginal discharge  Painful urination  Depression  Severe headache  Weakness  Any other( ) \| A  B  C  D  E  F  G  H  I  J \|  \|  \| **(E) Information on Utilization of maternal health services by wife** \| \| \| \| \| \| --- \| --- \| --- \| --- \| --- \| \| Sr.  no \| **Question and filter** \| **Coding Category** \|  \| Skip \| \| E.1 \| During last pregnancy your wife has gone for ANC check-up? \| Yes  No \| 1  2 \|  \| \| E.2 \| Where your wife delivered her last child? \| Home  Sub-center  Primary health center  Private hospital  Charity hospital  District hospital \| 1  2  3  4  5  6 \|  \| \| E.3 \| Did your wife had post natal check-ups? \| Yes  No \| 1  2 \|  \| | | | | |
